# Case Report: Prolonged thrombocytopenia after chemotherapy in solid tumors requiring long-term thrombopoietin receptor agonist support: a five-patient case series

**DOI:** 10.3389/fonc.2026.1924838

**Published:** 2026-09-07

**Authors:** Shengjie Wang, Yinan Yu, Lan Zhang, Qiuxia Liu

**Affiliations:** 1 Department of Oncology, Shaoxing People’s Hospital, The First Hospital of Shaoxing University, Shaoxing, Zhejiang, China; 2 Department of Hematology, Shaoxing People's Hospital, The First Hospital of Shaoxing University, Shaoxing, Zhejiang, China

**Keywords:** bone marrow, case series, chemotherapy-induced thrombocytopenia, megakaryopoiesis, prolonged thrombocytopenia after chemotherapy, solid tumors, thrombopoietin receptor agonist

## Abstract

Chemotherapy-induced thrombocytopenia (CIT) is a clinically significant complication of systemic anticancer therapy. Although CIT is commonly classified as nadir CIT or persistent CIT based on the timing and pattern of platelet recovery, prolonged thrombocytopenia after chemotherapy discontinuation remains poorly characterized. We report a retrospective case series of five patients with solid tumors who developed prolonged thrombocytopenia after chemotherapy discontinuation. Despite conventional supportive measures, platelet counts (PCs) failed to recover adequately; all patients subsequently received long-term thrombopoietin receptor agonist (TPO-RA) therapy to maintain PCs. TPO-RA supportive treatment was maintained for 7 to 22 months. Bone marrow (BM) examinations consistently demonstrated reduced or absent megakaryocytes, whereas overall hematopoietic activity ranged from relatively preserved to hypoplastic. At last follow-up, all patients were alive. No bleeding or thrombotic events were observed after discontinuation of anticancer therapy, and no clinically apparent TPO-RA-related adverse events were documented during treatment. One patient resumed anticancer therapy while receiving TPO-RA support, whereas the remaining four received no further anticancer treatment. This case series characterizes an understudied pattern of prolonged impairment of platelet recovery after chemotherapy that may require sustained thrombopoietic support. Larger prospective longitudinal studies are needed to clarify the underlying heterogeneity and identify patients most likely to require prolonged TPO-RA therapy.

## Introduction

Chemotherapy-induced thrombocytopenia (CIT) is a clinically significant complication of systemic anticancer therapy in patients with solid tumors (1). In a large US electronic health record study, the cumulative incidence of thrombocytopenia within 3 months after chemotherapy initiation in patients with solid tumors was 12.8%, with grade 3 and grade 4 thrombocytopenia occurring in 4.2% and 1.9% of patients, respectively (2). CIT may necessitate chemotherapy dose reduction, treatment delay, or treatment discontinuation and may increase bleeding risk, thereby compromising the planned delivery of anticancer therapy.

According to the timing and pattern of platelet decline and recovery, CIT is commonly classified into two major clinical patterns: nadir CIT and persistent CIT (1, 3). Nadir CIT refers to a transient decline in platelet count (PC) during a chemotherapy cycle followed by recovery before the next cycle is due (1). In contrast, persistent CIT is characterized by inadequate platelet recovery when the next treatment cycle is scheduled, preventing chemotherapy from proceeding safely at the planned dose and schedule; thrombocytopenia may consequently persist across multiple treatment cycles (1). Most available studies have focused on thrombocytopenia during active chemotherapy, including its incidence, severity, and immediate effects on treatment delivery. By contrast, the clinical course of thrombocytopenia that remains prolonged after chemotherapy discontinuation is poorly characterized, and the mechanisms underlying sustained impairment of platelet recovery and the optimal approach to long-term management remain uncertain.

Here, we report a case series of five patients with solid tumors who developed prolonged thrombocytopenia after chemotherapy. PCs failed to recover adequately despite conventional supportive treatment, and the patients were subsequently managed with long-term thrombopoietin receptor agonist (TPO-RA) therapy to maintain PCs. Through longitudinal clinical assessment, bone marrow (BM) evaluation, and analysis of treatment response, we describe differences in the clinical and marrow features of this patient group and discuss unresolved issues in diagnosis, potential mechanisms, and long-term management.

## Case presentation

### Clinical case 1: 80-year-old woman with ovarian cancer

**Figure 1:**
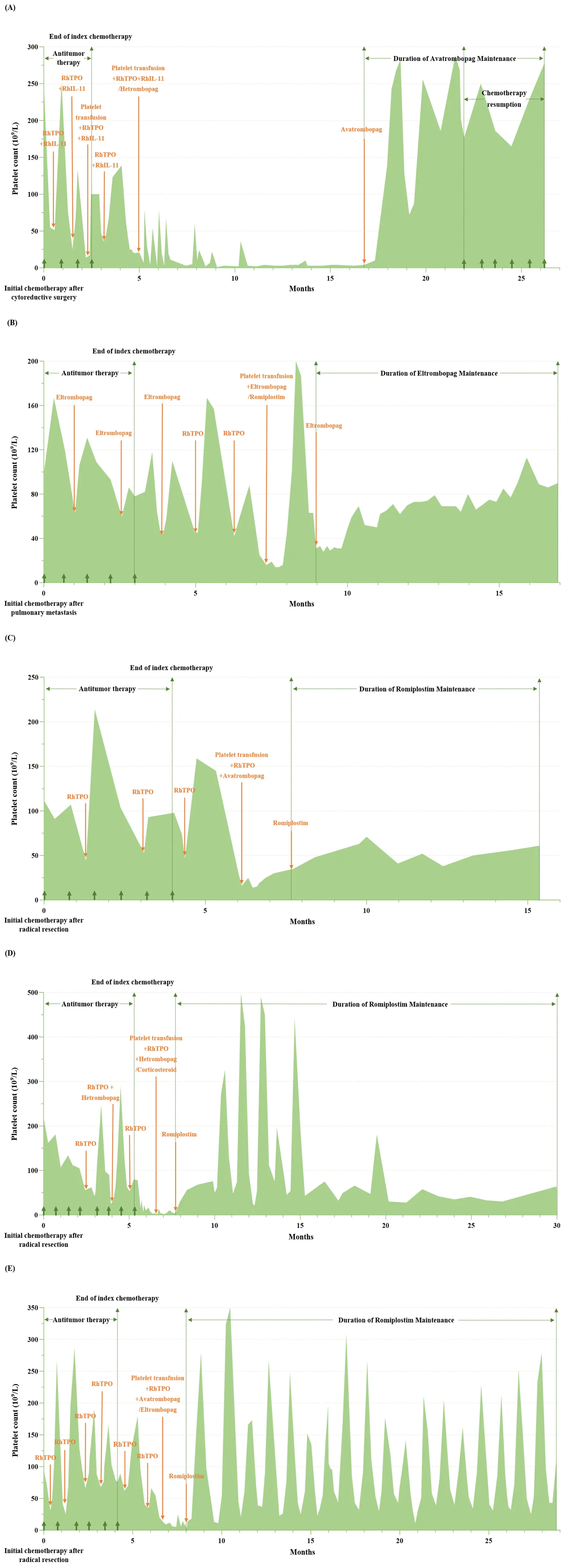
Longitudinal platelet trajectories and thrombopoietic support during and after the index chemotherapy course in five patients with prolonged thrombocytopenia. **(A)** Clinical Case 1, **(B)** Clinical Case 2, **(C)** Clinical Case 3, **(D)** Clinical Case 4, **(E)** Clinical Case 5. RhIL-11, recombinant human interleukin-11; RhTPO, recombinant human thrombopoietin.

The patient was diagnosed with advanced ovarian cancer. After two cycles of paclitaxel plus carboplatin (TC), she underwent cytoreductive surgery. At the start of cycle 3, the PC was 241×10^9^/L, and decreased to 51×10^9^/L after cycle 3; it subsequently increased to 252×10^9^/L after administration of recombinant human thrombopoietin (rhTPO) and recombinant human interleukin-11 (rhIL-11) (**Figure 1A**). After cycles 4 and 5, the PC declined to 25×10^9^/L and 14×10^9^/L, respectively, and subsequently returned to the normal range after platelet transfusion, rhTPO, and rhIL-11. Due to the occurrence of grade 4 thrombocytopenia according to the National Cancer Institute Common Terminology Criteria for Adverse Events (NCI-CTCAE), version 5.0 (4), the chemotherapy dose was reduced by 20% for cycle 6. Prolonged thrombocytopenia with recurrent grade 4 episodes continued for more than one year. Repeated platelet transfusion and administration of rhTPO, rhIL-11, and hetrombopag failed to stably maintain the PC. BM aspiration showed a moderate number of nucleated cells but no detectable megakaryocytes, whereas BM trephine biopsy showed active hematopoiesis without reticulin fibrosis (**Table 1**). Avatrombopag was initiated at 40 mg once daily. After approximately two weeks of treatment, the PC increased to 139×10^9^/L but declined again following discontinuation of avatrombopag. Due to tumor progression, the patient received six cycles of nanoparticle albumin-bound paclitaxel plus bevacizumab. At the last follow-up, the PC remained within the normal range during chemotherapy while the patient was receiving avatrombopag maintenance at 20 mg every other day; this maintenance therapy had been continued for 9 months.

**Table 1:** Clinical characteristics, bone marrow findings, and long-term TPO-RA maintenance in the five patients.

Case	Tumor type	Chemotherapy exposure	Key bone marrow findings	Maintaining treatment	TPO-RA maintenance duration	Follow-up duration*
1	Ovarian Cancer	TC×6	Moderate nucleated-cell cellularity with absent megakaryocytes; active hematopoiesis on biopsy; no reticulin fibrosis	Avatrombopag 20 mg every other day	9 months	23 months
2	Rectal Cancer	XELOX×6 → XELOX+BEV×5	Markedly reduced nucleated cells with rare megakaryocytes; active hematopoiesis on biopsy; no reticulin fibrosis	Eltrombopag 25 mg every other day	8 months	14 months
3	Urothelial Carcinoma	GC×6	Hematopoietic hypoplasia with rare megakaryocytes; no reticulin fibrosis	Romiplostim 250 μg once weekly	7 months	11 months
4	Sigmoid Colon Cancer	XELOX×8	Active proliferation of nucleated cells with rare megakaryocytes; trephine biopsy not performed	Romiplostim 250 μg approximately once monthly**	22 months	24 months
5	Gastric Cancer	SOX×2 → XELOX×1 → FOLFOX×3	Scant nucleated cells with absent megakaryocytes; trephine biopsy not performed	Romiplostim 250 μg once monthly	20 months	24 months

*Follow-up duration was calculated from cessation of the index chemotherapy course associated with prolonged thrombocytopenia to the last available follow-up.

**In Case 4, the interval of romiplostim administration was adjusted according to platelet count and was approximately once monthly during long-term maintenance. TPO-RA, thrombopoietin receptor agonist; TC, paclitaxel plus carboplatin; XELOX, oxaliplatin plus capecitabine; BEV, bevacizumab; GC, gemcitabine plus carboplatin; SOX, S-1 plus oxaliplatin; FOLFOX, folinic acid, fluorouracil, and oxaliplatin.

### Clinical case 2: 73-year-old man with rectal cancer

The patient underwent radical resection for rectal cancer followed by six cycles of oxaliplatin plus capecitabine (XELOX). During adjuvant therapy, grade 2 thrombocytopenia occurred, with a nadir PC of 67×10^9^/L. Corticosteroids were administered specifically for the thrombocytopenia, and the PC subsequently returned to the normal range. After pulmonary metastases developed, the patient received five cycles of XELOX plus bevacizumab. At the start of retreatment, the PC was 101×10^9^/L (**Figure 1B**). After cycles 2 and 4, grade 2 thrombocytopenia occurred, with a nadir PC of 67×10^9^/L; eltrombopag was administered, and the PC recovered within one week. Thrombocytopenia recurred after cycle 5. The PC increased after eltrombopag administration but decreased again after treatment discontinuation. Subsequent rhTPO treatment failed to maintain a stable PC after withdrawal. During this period, the PC decreased to 16×10^9^/L, accompanied by right conjunctival hemorrhage. BM aspiration showed markedly reduced nucleated cells and rare megakaryocytes; BM trephine biopsy showed active hematopoiesis without reticulin fibrosis (**Table 1**). After treatment with platelet transfusion, eltrombopag 50 mg once daily, and a single dose of romiplostim, the PC increased to 101×10^9^/L after three weeks but declined after temporary discontinuation of eltrombopag. At the last follow-up, eltrombopag 25 mg every other day had maintained the PC for 8 months.

### Clinical case 3: 60-year-old patient with urothelial carcinoma

The patient underwent radical nephroureterectomy with bladder cuff excision for a malignant ureteral tumor, followed by four cycles of intravesical gemcitabine and six cycles of gemcitabine plus carboplatin (GC). At the start of GC, the PC was 111×10^9^/L (**Figure 1C**). After cycles 2 and 4, it decreased to 44×10^9^/L and 53×10^9^/L, respectively, and subsequently increased after rhTPO administration. After cycle 6, it decreased to 47×10^9^/L and increased to 159×10^9^/L after rhTPO, followed shortly thereafter by a sharp decline to 16×10^9^/L. Platelet transfusion, rhTPO, and avatrombopag failed to maintain a stable PC, and treatment was switched to romiplostim. The PC increased but declined after romiplostim discontinuation. BM aspiration and trephine biopsy showed hematopoietic hypoplasia with rare megakaryocytes; reticulin staining was negative (**Table 1**). At the last follow-up, romiplostim 250 μg once weekly had been continued for 7 months, with PCs maintained at 40–65×10^9^/L.

### Clinical case 4: 53-year-old patient with sigmoid colon cancer

The patient underwent radical resection for sigmoid colon cancer followed by four cycles of full-dose XELOX. On day 12 of cycle 4, the PC was 56×10^9^/L (**Figure 1D**) and subsequently increased to 118×10^9^/L after rhTPO administration. Two cycles of dose-reduced XELOX were then administered. Grade 4 thrombocytopenia occurred after cycle 6, followed by PC recovery after rhTPO and hetrombopag administration. Chemotherapy doses were further reduced during cycles 7 and 8, after which thrombocytopenia persisted. BM aspiration showed active proliferation of nucleated cells with rare megakaryocytes; BM trephine biopsy was not performed (**Table 1**). Platelet transfusion, rhTPO, hetrombopag, and corticosteroids specifically administered for thrombocytopenia were not followed by sustained platelet recovery. After treatment was switched to romiplostim, the PC gradually increased but declined again after treatment discontinuation. At the last follow-up, the PC was maintained at 30–75×10^9^/L with romiplostim 250 μg administered approximately once monthly for 22 months.

### Clinical case 5: 73-year-old patient with gastric cancer

The patient underwent radical gastrectomy for gastric cancer followed by two cycles of oxaliplatin plus S-1 (SOX). After cycles 1 and 2, the PC decreased to 31×10^9^/L and 25×10^9^/L, respectively (**Figure 1E**), and subsequently returned to the normal range after rhTPO administration. Due to poor appetite and thrombocytopenia, the regimen was changed to XELOX for cycle 3. On day 14 of cycle 3, the PC was 66×10^9^/L and subsequently increased to 112×10^9^/L after rhTPO. Severe diarrhea and thrombocytopenia during capecitabine treatment prompted a change to three cycles of dose-reduced folinic acid, fluorouracil, and oxaliplatin (FOLFOX). Thrombocytopenia persisted after cycle 6, with a nadir PC of 4×10^9^/L. BM aspiration yielded scant nucleated cells, with no megakaryocytes detected; BM trephine biopsy was not performed (**Table 1**). Platelet transfusion, rhTPO, avatrombopag, and eltrombopag failed to maintain a stable PC. The PC subsequently normalized after switching to romiplostim but declined after discontinuation. At the last follow-up, romiplostim 250 μg once monthly had been continued for 20 months, and the PC remained stable.

### Overall follow-up

At the last follow-up, all five patients were alive. TPO-RA maintenance therapy had been continued for 9 months in Case 1, 8 months in Case 2, 7 months in Case 3, 22 months in Case 4, and 20 months in Case 5. No bleeding or thrombotic events were observed after discontinuation of anticancer therapy, and no clinically apparent adverse events considered related to TPO-RA, rhTPO, or rhIL-11 were documented during treatment. During follow-up, leukocyte and hemoglobin abnormalities resolved, and no clinical diagnosis of therapy-related myelodysplastic syndrome or acute myeloid leukemia was made during the available follow-up period. Serial bone marrow examinations were not routinely performed. Case 1 resumed anticancer therapy while receiving TPO-RA maintenance therapy, whereas Cases 2–5 received no further anticancer treatment.

## Discussion

The incidence and severity of CIT vary substantially among patients and are influenced by tumor type, chemotherapy regimen and dose intensity, cumulative treatment exposure, baseline hematopoietic reserve, and prior anticancer therapy (3, 5, 6). Differences in CIT risk across treatment settings likely reflect the interaction between chemotherapy exposure and individual susceptibility to hematopoietic injury. Accordingly, CIT should not be regarded as a uniform treatment-related toxicity.

Current management is guided by the severity and clinical pattern of CIT (3). Platelet transfusion remains an important short-term supportive measure, including prophylactic transfusion at PCs <10 × 10^9^/L and transfusion in settings such as active bleeding or invasive procedures when a rapid increase in PC is required (3). However, platelet transfusion has important limitations for long-term support because the platelet increment is transient and variable, donor platelet availability is limited, and repeated transfusions increase the risks of transfusion reactions, alloimmunization, and subsequent platelet transfusion refractoriness (7). The clinical use of recombinant human interleukin-11 (rhIL-11) has been substantially limited by toxicity and tolerability concerns, whereas recombinant human thrombopoietin (rhTPO) is used predominantly in China and selected regions; its use in this series therefore reflects regional real-world practice (3, 6). In this series, thrombopoietic treatment was individualized according to the severity and clinical consequences of thrombocytopenia and, during active chemotherapy, the need to facilitate subsequent anticancer treatment. Selection and modification of individual agents also reflected practical considerations in real-world care, including treatment accessibility, cost, route of administration, and patient adherence.

Although no TPO-RA is currently approved specifically for CIT by the US Food and Drug Administration or the European Medicines Agency, TPO-RAs have emerged as a potential therapeutic option for patients with CIT requiring sustained thrombopoietic support. Importantly, the early use of thrombopoietic agents during chemotherapy-associated platelet nadirs in Cases 1 and 2 should be distinguished from their later use for prolonged thrombocytopenia after chemotherapy. In particular, the rapid platelet recovery observed within one week in Case 2 cannot be reliably attributed to eltrombopag, because spontaneous hematologic recovery after chemotherapy may have contributed substantially. Romiplostim has a comparatively substantial clinical evidence base. In a prospective randomized study, 93% of patients treated with romiplostim achieved platelet correction within 3 weeks (8). More recently, the phase III RECITE randomized placebo-controlled trial provided high-level evidence supporting romiplostim for persistent CIT (9). Other TPO-RAs, including avatrombopag and eltrombopag, have also been evaluated, although the sample sizes, study designs, and clinical endpoints differ across agents (10–12). Switching between thrombopoietic agents may represent an additional strategy when platelet recovery is inadequate, a response cannot be maintained, or treatment-related factors preclude continued use of the initial agent. Direct evidence for switching in CIT remains limited, although a recent multicenter real-world study has described avatrombopag switching after failure or loss of response to other thrombopoietic agents in severe persistent cancer treatment-induced thrombocytopenia (13). Experience with TPO-RA switching in immune thrombocytopenia provides additional clinical context (14, 15). Whether switching improves the durability of response in prolonged thrombocytopenia after chemotherapy requires further study.

BM examinations in this series consistently demonstrated reduced or absent megakaryocytes, whereas overall hematopoietic activity ranged from relatively preserved to hypoplastic. Marked megakaryocyte reduction with relative preservation of other hematopoietic lineages raised the possibility of an acquired amegakaryocytic thrombocytopenic purpura (AATP)-like phenotype in some patients (16), whereas hypoplastic marrow findings were more compatible with broader chemotherapy-related marrow injury. The absence of compensatory megakaryocytic proliferation made mechanisms dominated by peripheral platelet destruction, such as immune thrombocytopenia or drug-induced immune thrombocytopenia, less typical; however, the available data were insufficient to establish a definitive pathological classification. In addition, BM examinations were performed at different intervals after chemotherapy exposure and may therefore have captured different stages of marrow injury and hematopoietic recovery. Thus, the observed differences may reflect distinct patterns of hematopoietic injury, different stages within a dynamic recovery process, or both.

Chemotherapy-related hematopoietic injury can occur at multiple levels relevant to platelet production, including hematopoietic stem and progenitor cells, megakaryopoiesis, and the BM microenvironment that supports hematopoietic recovery (17–19). Although hematopoietic suppression after chemotherapy is often at least partly reversible, cumulative or repeated exposure may result in more prolonged impairment of hematopoietic recovery (19, 20). Accordingly, the prolonged thrombocytopenia and heterogeneous marrow findings observed in this series may reflect different degrees or combinations of megakaryocytic suppression and broader marrow dysfunction rather than a single pathological mechanism.

Several limitations should be considered. This retrospective, single-center case series included only five clinically heterogeneous patients with differences in tumor type, chemotherapy exposure, marrow findings, and treatment history; therefore, direct comparisons across cases are limited and the findings require validation in larger prospective cohorts. Serial BM assessments and comprehensive mechanistic investigations were not routinely performed, limiting our ability to determine whether the observed marrow differences represent distinct patterns of hematopoietic injury or different stages of recovery. The absence of routine serial marrow surveillance also limited definitive exclusion of late therapy-related myeloid neoplasms. In addition, this study focused on patients with prolonged thrombocytopenia after chemotherapy who required prolonged thrombopoietic support; therefore, the findings should not be generalized to all patients with CIT. Nevertheless, the longitudinal clinical courses, treatment responses, and BM findings provide additional characterization of this insufficiently studied patient population.

Future studies should determine whether prolonged thrombocytopenia after chemotherapy encompasses different patterns or stages of impaired thrombopoiesis. Prospective longitudinal studies integrating platelet kinetics, treatment exposure, serial marrow characteristics, and mechanistic biomarkers may help clarify these differences and identify predictors of prolonged thrombopoietic support requirements and sustained TPO-RA response. Such information may improve patient selection and facilitate more individualized management of prolonged thrombocytopenia after chemotherapy.

## Data Availability

The original contributions presented in the study are included in the article/supplementary material. Further inquiries can be directed to the corresponding author.
